# Sleep Quality Moderates the Association Between Routine Disruption Stress and Affect in Midlife and Late Adulthood

**DOI:** 10.1093/geroni/igaf122.1918

**Published:** 2025-12-31

**Authors:** Eunjin Tracy, Jichan Kim, Pei-Shu Chao, Shadi Ansari, Eunjung Kim

**Affiliations:** University of Missouri, Columbia, Missouri, United States; Liberty University, Lynchburg, Virginia, United States; University of Missouri, Columbia, Missouri, United States; University of Missouri, Columbia, Missouri, United States; University of Missouri, Columbia, Missouri, United States

## Abstract

Disruptions in daily routines can misalign circadian rhythms, reduce structure and predictability, and contribute to emotional dysregulation. Individuals with poor sleep quality may be particularly vulnerable to these stressors due to preexisting circadian misalignment, yet few studies have examined these associations at the daily level or the potential role of sleep in these relationships. This study analyzed data from 1,011 participants (M age = 55.34, range = 34–84) in the Midlife in the United States (MIDUS) Daily Stress Project, assessing daily stress related to routine disruptions and affect over eight days, along with global sleep quality. Multilevel modeling revealed significant within- and between-person effects: higher stress related to daily routine disruption was associated with greater negative affect and lower positive affect at both within- and between-person levels. Sleep quality significantly moderated these associations for negative affect at both levels. At the within-person level, routine disruption stress increased negative affect only among those with poor sleep quality (z = 4.49, p < .001 vs. z = 1.12, p = .26 for better sleep quality). At the between-person level, the association between routine disruption stress and negative affect was stronger for those with poorer sleep quality (z = 7.37, p < .001) compared to those with better sleep quality (z = 3.58, p < .001). These findings underscore the role of daily routines in emotional regulation in midlife and suggest that better sleep quality may buffer against stress-related affective disturbances. Interventions targeting routine stability and sleep quality may enhance emotional well-being.

